# Astrocytosis in parkinsonism: considering tripartite striatal synapses in physiopathology?

**DOI:** 10.3389/fnagi.2014.00258

**Published:** 2014-09-24

**Authors:** Giselle Charron, Evelyne Doudnikoff, Marie-Helene Canron, Qin Li, Céline Véga, Sebastien Marais, Jérôme Baufreton, Anne Vital, Stéphane H. R. Oliet, Erwan Bezard

**Affiliations:** ^1^Institut des Maladies Neurodégénératives, Université de Bordeaux, UMR 5293Bordeaux, France; ^2^CNRS, Institut des Maladies Neurodégénératives, UMR 5293Bordeaux, France; ^3^Institute of Laboratory Animal Sciences, Chinese Academy of Medical Science and Peking Union Medical CollegeBeijing, China; ^4^UFR Sciences de la Vie, University Pierre et Marie Curie (UPMC)Paris, France; ^5^Bordeaux Imaging Center, UMS 3420, Université de BordeauxBordeaux, France; ^6^CNRS, Bordeaux Imaging Center, UMS 3420Bordeaux, France; ^7^INSERM, Bordeaux Imaging Center, US 004Bordeaux, France; ^8^Neurocentre Magendie, U862, Institut National de la Santé et de la Recherche MédicaleBordeaux, France

**Keywords:** immunohistochemistry, medium spiny neuron, astrocyte, dopamine, mouse, rat, monkey, human

## Abstract

The current concept of basal ganglia organization and function in physiological and pathophysiological conditions excludes the most numerous cells in the brain, i.e., the astrocytes, present with a ratio of 10:1 neuron. Their role in neurodegenerative condition such as Parkinson’s disease (PD) remains to be elucidated. Before embarking into physiological investigations of the yet-to-be-identified “tripartite” synapses in the basal ganglia in general and the striatum in particular, we therefore characterized anatomically the PD-related modifications in astrocytic morphology, the changes in astrocytic network connections and the consequences on the spatial relationship between astrocytic processes and asymmetric synapses in normal and PD-like conditions in experimental and human PD. Our results unravel a dramatic regulation of striatal astrocytosis supporting the hypothesis of a key role in (dys) regulating corticostriatal transmission. Astrocytes and their various properties might thus represent a therapeutic target in PD.

## Introduction

Dopamine (DA) deprivation in Parkinson’s disease (PD; Ehringer and Hornykiewicz, 1960) provokes complex and multiple changes in striatal DA target neurons that contribute to development of characteristic motor disorders such as bradykinesia, rigidity and resting tremor (Calabresi et al., 2000; Nicola et al., 2000). The mechanisms triggering and/or contributing to the morphological and functional modifications affecting the basal ganglia (BG) network remain elusive (Gerfen and Surmeier, 2011; Picconi et al., 2012). Puzzlingly enough, the role of astrocytes in such complex pathological changes has almost remained untouched. The current concept of BG organization and function excludes the most numerous cells in the brain (Tsacopoulos and Magistretti, 1996). For decades, astrocytes have been regarded as passive partners of neurons in the central nervous system, but this view has been challenged and they are now integrated in the concept of “tripartite synapse”. Indeed astrocytes have a multitude of processes that are intertwined within the neuropil ensheathing synaptic contacts, they possess receptors and reuptake sites for neurotransmitters, so they sense and integrate synaptic activity. They are essential for the metabolic support of neurons by promoting neurovascular coupling and for neurotransmitters synthesis. Moreover, depending on intracellular Ca^2+^ levels, they may release signaling molecules known as gliotransmitters that have feedback actions on neurons (Perea et al., 2009; Parpura et al., 2012; Santello et al., 2012). Astrocytes overlap little with each other, forming a tiling organization that ensures that a given set of synapses is covered by only one astroglial cell. One unique feature of astrocytes is their high level of intercellular connectivity mediated by connexins (Cx), the proteins forming gap junction (GJ) channels, of which the Cx 43 and 30 are the main species in the adult brain (Dermietzel et al., 1991; Nagy et al., 1999). Cx are embedded in the plasma membrane and, when associated head-to-head between two neighboring cells, form a GJ. Cx can also operate as hemichannels, allowing exchange of molecules between the cytoplasmic and extracellular media (Hofer and Dermietzel, 1998). The central pore of hemichannels or GJs allows the passage of ions and small molecules allowing an ionic, biochemical or metabolic coupling with neighboring cells.

It is likely that if so many important brain functions depend on appropriate astrocyte-neuron interactions, some pathological situations might be caused by a dysfunction or failure in this process at one level or another. This could be the case in PD where astrocytes have been shown to play a crucial role in the initiation and progression of the disease (Lee et al., 2010; Halliday and Stevens, 2011; Sathe et al., 2012) as the distribution of α-synuclein-positive astrocytes parallels the distribution of Lewy bodies (Braak et al., 2007). Moreover loss of DA neurons in the substantia nigra pars compacta is knowingly associated with an increase number of activated astrocytes (Hirsch et al., 2003; Gomide and Chadi, 2005; Mcgeer and Mcgeer, 2008). Astrocytosis in PD was so far primarily approached from the neurodegeneration angle but its role might well go beyond these effects. Before embarking into physiological investigations of the yet-to-be-identified “tripartite” synapses in the basal ganglia in general and the striatum in particular, we therefore characterized anatomically the PD-related modifications in astrocytic morphology, the changes in astrocytic network connections and the consequences on the spatial relationship between astrocytic processes and asymmetric synapses in normal and PD-like conditions in experimental and human PD. Our results unravel a dramatic regulation of striatal astrocytosis supporting the hypothesis of a key role in (dys) regulating corticostriatal transmission. Astrocytes and their various properties might thus represent an interesting therapeutic target in PD.

## Materials and methods

### Rodent material

Experiments were performed in accordance with French (87-848, Ministère de l’Agriculture et de la Forêt) and European Communities Council Directive (2010/63/EU) for care of laboratory animals and were approved by the Ethical Committee of Center National de la Recherche Scientifique, Région Aquitaine (5012099-A).

#### 6-hydroxydopamine (6-OHDA) rats

Adult male Sprague Dawley rats (Charles River Laboratories) weighing 175–200 g were sham operated and 6-OHDA-lesioned (*n* = 4/group) with injection (3 μg/μl) in the right medial forebrain bundle (2.5 μl at anteroposterior = −3.7 mm, mediolaterial = +1.7 mm and dorsoventral = −8 mm, relative to bregma) after pretreatment with citalopram (1 mg/kg i.p.; Lundbeck), an inhibitor of serotonin reuptake, and with desipramine hydrochloride (20 mg/kg i.p.; Sigma-Aldrich), an inhibitor of noradrenergic reuptake, as previously described (Charron et al., 2011; Porras et al., 2012). A >95% loss of tyrosine hydroxylase (TH)-immunopositive fibers in the striatum was required for inclusion. Animals were terminated 30 days post-surgery.

#### 6-OHDA mice

45-day-old laboratory-bred adult male C57BL/6 mice weighting 30 ± 3 g received unilateral stereotaxic intra right medial forebrain bundle injection of vehicle (1 μl) or 6-OHDA (3 μg/μl) (*n* = 5/group) at the following coordinates according to the mouse brain atlas: AP −0.7; L −1.2; DV −4.7 as previously described (Fasano et al., 2010). On day 21, all animals were sacrificed and severity of DA denervation was assessed by analyzing striatal levels of TH.

#### Reserpine mice

45-day-old laboratory-bred adult male C57BL/6 mice weighting 30 ± 3 g were injected with either saline (controls) or a combination of 2.5 mg/kg reserpine i.p. (Sigma Aldrich) at 24 h and 100 mg/kg of α-methyl-*p*-tyrosine (Sigma Aldrich) (*n* = 4/group) 24, 16, 4 and 1 h before sacrifice, as previously described (Berthet et al., 2012).

#### Terminal procedure

Animals were deeply anesthetized with chloral hydrate (150 mg/kg i.p., VWR) and transcardially perfused with 2% paraformaldehyde (PFA) and 0.2% glutaradehyde. Brains were post-fixed overnight in 2% PFA at 4°C. Coronal sections were cut at 50 μm on a Vibratome (Leica, VT 1000S, Wetzlar, Germany) and collected in phosphate buffer saline (PB saline (PBS) pH 7.4). To enhance the penetration of immunoreagents, the sections were equilibrated in a cryoprotectant solution, PBS with 25% saccharose, freeze thawed in isopentane and stored in PBS with 0.03% sodium azide at 4°C.

### Non-human primate material

All experiments were carried out in accordance with the European Communities Council Directive (2010/63/EU) for care of laboratory animals in an AAALAC-accredited facility and were approved by the Institute of Lab Animal Science IACUC. Animals were housed in individual primate cages under controlled conditions of humidity (50 ± 5%), temperature (24 ± 1°C) and light (12 h light/12 h dark cycles, time lights on 8:00 am), food and water were available *ad libitum* and animal care supervised by veterinarians skilled in the healthcare and maintenance of non-human primates. Six female macaca mulatta monkeys (Xierxin, Beijing, PR of China), previously presented in Fernagut et al. (2010), Santini et al. (2010) and Porras et al. (2012), were either kept normal (control, *n* = 3) or intoxicated with 1-methyl-4-phenyl-1,2,3,6-tetrahydropyridine (MPTP) hydro­chloride (MPTP, *n* = 3). Bilateral parkinsonian syndrome had stabilized for 6 months without any dopaminergic supplementation before termination. Animals were deeply anesthetized with sodium chloral hydrate (150 mg/kg) and perfused transcardially with 4% PFA in phosphate buffer (PB, 0.1 M). Brains were removed, bisected along the midline, stored in 2% PFA overnight at 4°C, and cut into 60 μm frontal sections with vibratome (Leica, VT1000S, Wetzlar, Germany). Sections were collected in PBS, cryoprotected in PBS with 25% saccharose, freeze-thawed in isopentane and stored in PBS with 0.03% sodium azide until use. The clinical assessments and the characterization of the extent of nigrostriatal denervation have been previously published (Fernagut et al., 2010; Santini et al., 2010; Porras et al., 2012) showing that all the MPTP-treated animals displayed comparable lesion of the nigrostriatal pathway.

### Post-mortem human samples

The observations on human tissue were based on the analysis of formalin fixed and paraffin embedded human specimens from an archival collection (Comité Protection des Personnes No. CEBH 2009/03; Ministère Enseignement Supérieur et Recherche: DC-2008-337) declared and approved by the ethics committee (“Comité de Protection des Personnes Sud-Ouest et Outre Mer III”) of Bordeaux University Hospital (Charron et al., 2011). Striatum material was available for both neuropathologically-confirmed cases of PD (*n* = 3; 2 males, 1 female; 62 ± 7 years old at death) and controls with no detectable central nervous system disease (*n* = 3; 2 males, 1 female; 63 ± 8.5 years old at death) (Charron et al., 2011). The post-mortem delay was similar for all cases (<24 h) but the female PD case with 60 h. The striatum was studied within a coronal 5 mm-thick section demonstrating connections between the head of the caudate and the putamen across the internal capsule.

## Immunohistochemical procedures

### Immunoperoxydase labeling on animal material

The number of striatal and globus pallidus (GP) astrocytes were defined after immunolabeling with the peroxydase technique (Charron et al., 2011). Sections were incubated overnight at room temperature (RT) in a combination of two primary antibodies directed against two complementary markers of astrocytes, i.e., Glial fibrillary acidic protein (GFAP; MAB3402, Millipore or Z03334, Dako) and S100β (PAB11341, Abnova or Ab7852, Abcam), a calcium-binding protein synthesized in, and constitutively secreted, by astrocytes (Van Eldik and Zimmer, 1987; Sorci et al., 2010). This was then incubated in biotinylated secondary antibody and then in avidin-biotin complex (ABC; Vectastain Elite ABC kit). Immunoreactivity was revealed with Novared kit (Vector Laboratories).

### Immunoperoxydase labeling on human samples

4 μm-thick paraffin-embedded striatal sections were processed as previously described (Vital et al., 2009; Charron et al., 2011). Briefly, sections were dewaxed, pressure-cooked in sodium citrate buffer (0.1 M, pH 6.0), washed, blocked with a universal blocking buffer (Biogenex) and incubated with GFAP antibody overnight. Control sections were incubated with blocked antiserum. After transfer in 3% H_2_O_2_/PBS, the sections were treated with a ready-to-use goat anti rabbit En Vision-HRP enzyme conjugate (Dako, Trappes, France) for 30 min. The color was developed using the highly sensitive diaminobenzidine plus (DAB+) (Dako, Trappes, France). Sections were counterstained with Mayer’s hemalum and mounted with an aqueous agent for microscopy (aquatex; Merck).

### Immunofluorescent labeling

Astrocytes were detected by fluorescent immunohistochemistry using anti-GFAP, anti-S100β or anti-Glutamate Transporter (GLT1; AB1783, Millipore) antibodies (Chaudhry et al., 1995; Lehre et al., 1995). Sections were incubated with Normal donkey serum (NDS) containing 0.05% tween 20 for 45 min and then in primary antibody supplemented with 1% NDS overnight at RT. After thorough washing, sections were incubated for 90 min at RT in secondary antibody conjugated to alexa 568 fluorochrome (1:400 Invitrogen).

### Immunofluorescent double-labeling

Those experiments aimed at studying the distribution and the composition of GJs connecting the astrocytes to form the astrocytic network (Giaume et al., 2010). GJs are formed of Cx of various molecular weights, e.g., Cx30 (728, Invitrogen) or Cx43 (MAB3068, Millipore). Dual labeling of GFAP and any Cx was achieved as described above except that two primary antibodies derived from different animal species were applied together. Similarly, two secondary antibodies raised in different species and labeled with two fluorochromes, alexa 488 against rabbit and alexa 568 against mouse, were applied.

### Immunoperoxidase labeling at electron microscopy level

GLT1 used as astrocyte marker was detected at subcellular level. Sections were incubated in 4% normal goat serum (NGS) for 45 min, then in GLT1 antibody (1:1000) supplemented with 1% NGS overnight at RT and finally incubated for 90 min at RT in biotinylated goat anti-guinea pig (1:200, Jackson Immuno Research). After rinsing, ABC (1:200 in PBS, Vectastain Elite ABC kit, Vector laboratories, Burlingame, CA) for 90 min at RT. Peroxidase activity was revealed by the glucose oxidase- DAB-nickel method (Shu et al., 1988). Negative immunohistological control demonstrated the absence of signal when omitting the first antibody. The sections were rinsed, post-fixed in 1% osmium tetroxide and dehydrated in ascending series of ethanol dilutions that also included 70% ethanol containing 1% uranyl acetate. They were then treated with propylene oxide, impregnated in resin overnight (Durcupan ACM; Fluka, Buchs, Switzerland), mounted on glass slides and cured at 60°C for 48 h. Areas of interest were cut out from the sections and glued to blank cylinders of resin. Ultrathin sections were collected on pioloform-coated single slot copper grids.

### Immunogold labeling

GLT1 and Cx30 were analyzed at the electron microscopic level specifically in striatum by the pre-embedding immunogold technique as previously described (Dumartin et al., 1998, 2000; Guigoni et al., 2007; Berthet et al., 2009). Sections were incubated in 4% NDS for 45 min and then in GLT1 (1:1000) or Cx30 (1:500) for 48 h at 4°C. After washing in PBS, the sections were incubated for 2 h at RT in biotinylated donkey anti-rabbit (1:200 GE Healthcare UK) or biotinylated goat anti-guinea pig (1:200 Jackson). After rinsing in (PBS, 2% BSAc, 0.2% Gel), sections were incubated in Nanogold streptavidine (1:100 in PBS BSAc Gel) for 3 h. The sections were then washed and post- fixed in 1% glutaraldehyde for 10 min. after rinsing in H_2_O, the signal of the immunoparticles was increased using a silver enhancement kit (HQ Silver, Nanoprobes, USA).

## Image analysis

Fluorescent images were captured on a confocal Leica microscope DM 2500 TCS SPE. Digital images were acquired separately for each wavelength [488 nm (green) and 568 nm (red)] and then merged. Any adjustments to brightness and contrast were made uniformly to all parts of the image. Cell counting and measurement of astrocyte diameter were performed using a computerized image analysis system (Mercator V6.50, Explora Nova) linked to a Leica microscope type DM 6000B (x20 objective). Quantitative analysis was carried out on the whole striatum or GP areas on at least three sections per animal and results were expressed as an average density of astrocyte per μm^2^. To minimize the inherent variability in the immunochemical procedures, comparisons were made in tissue sections that were processed simultaneously. Only those astrocytes that were on the surface of the sections, where antibody penetration was guaranteed, were counted. The length of astrocytic processes was defined as the distance between the soma and the detectable end of an extended process identified by GFAP staining. Astrocyte diameter was determined by measuring the longest axis of the cell body through the nucleus.

### Definition of astrocyte surface area and connexin repartition

Tissue sections were examined with a confocal Leica microscope DM 2500 TCS SPE. Z-axis image stacks were acquired (37 images, 0.3 μm Z-step size) using the optimal pixel size relative to the objective used (HCX PL Apo CS 63X). Imaris software (Bitplane AG, Zurich Switzerland) was used to create 3-D reconstruction and morphometric measurements. The surface function was used to reconstruct GFAP-labeled cell bodies and processes of astrocytes and evaluate the covered area within a given volume. The spot module was used to define the pinpointed location of Cx with red point objects and numerate immunopositive spots.

### Definition of astrocyte process repartition at subcellular level

The analysis of the number of asymmetrical synapses was performed on digital images obtained with a computer-linked CCD camera (Gatan) mounted on Hitachi H-7650 electron microscope at a final magnification of 15000 using the Metamorph software (version 4.6r5, Universal Imaging, Paris, France). GLT1 positive astrocytic processes surrounding asymmetric synapses were considered as GLT1 positive complex. To eliminate artifactual differences in the numbers of labeled profiles, an issue classically related to the limited penetration of immunoreagents, electron microscopic images were examined only from thin sections collected from the most superficial vibratome sections. The measures were performed on four animals per group on a minimum of 30 randomly selected dendritic fields. A dendritic field is defined as an area that does not contain a cell body. Indeed, given the difference in size between cell body and dendrites, the presence of a cell body in the randomly selected counting area would imbalance the counting procedure. 1900 asymmetric synapses were observed in 130 dendritic fields. The results are expressed as the proportion of GLT1 positive or GLT1 negative synaptic complex present in the striatum of each individual.

## Results

### Astrocyte morphology in control animals and human

**Figure 1:**
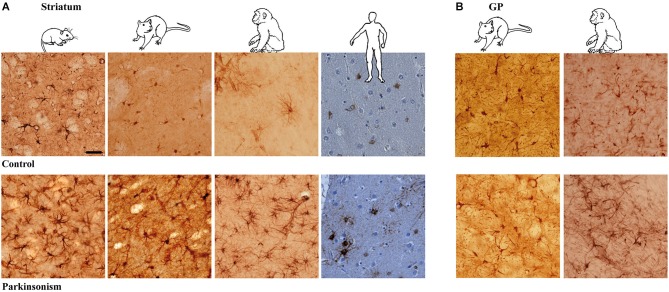
**Astrocytic reaction to dopamine depletion in striatum and GP of animal models of PD and parkinsonian patients**. Dopamine depletion increase GFAP-S100β immunolabeing in striatum **(A)** and GP **(B)** of reserpine mouse, 6-OHDA rat, MPTP non-human primate models of PD and PD patients (scale bar = 30 μm).

Immunodetection of astrocytes requires using at least two co-localizing markers, namely GFAP and S-100β, as they label distinct components of the astrocytes (Boyes et al., 1986; Raponi et al., 2007). S-100β locates mainly within astrocytic cytoplasm while GFAP reveals the cytoskeletal structure and delineates the glial processes, especially of activated astroglial cells, with the limit of labeling only the structure of primary branches, i.e., up to 15% of the total astrocyte volume (Bushong et al., 2002). In addition, S-100β expression in GFAP-positive cells defines a mature astrocytic stage (Raponi et al., 2007). Astrocyte morphology is strikingly similar across species (mouse, rat, macaque and human) although clearly different between the striatum (Figure 1A) and the GP (GP in rodents, GP pars externalis—GPe—in primates; Figure 1B). While striatal GFAP-S-100β positive cells are protoplasmic astrocytes with round somata and numerous short, thin and ramified processes (Figure 1A), GP-positive cells are certainly protoplasmic astrocytes as well but the majority present a roughly fusiform shape. In both structures, and whatever the species, astrocytes are grouped in close vicinity of capillaries.

### Astrocyte morphology in experimental parkinsonism and PD

**Figure 2:**
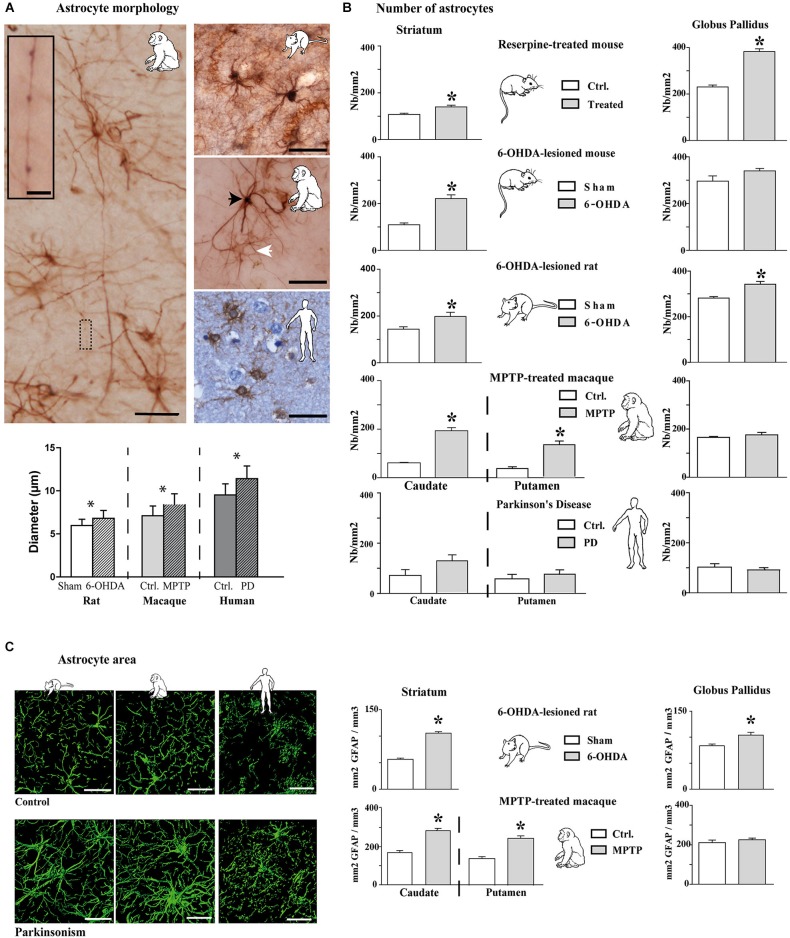
**Morphological and quantitative analysis of astrocytic reaction to dopamine depletion in striatum and GP of animal models of PD and parkinsonian patients. (A)** GFAP-S100β immunostaining enables demonstrating significant increase in astrocyte soma diameter in the rat and monkey models of PD (*n* = 4 sham and 4 6-OHDA rats; *n* = 3 control and 3 MPTP monkeys; mean ± SEM) (scale bar = 30 μm but in inset, 10 μm). Primates show two level of immunoreactivity indicated by a black arrowhead for intense labeling and a white arrowhead for moderate staining. **(B)** Quantitative analysis of astrocyte number in striatum and GP in controls and in animal models of PD (*n* = 4 reserpine mouse, *n* = 5 6-OHDA mouse, *n* = 4 6-OHDA rat, *n* = 3 MPTP non-human primate) as well as in parkinsonian patients (*n* = 3) showing increase in astrocyte number. * denotes a significant difference (Student *t*-test; *p* < 0.05). (C) Area analysis of astrocytic processes after GFAP immunostaining and 3D reconstruction. Left: representative examples of GFAP immunostaining in the striatum of 6-OHDA rat and MPTP monkey models of PD as well as in PD patients (Scale bar = 30 μm). Right: Astrocytic area significantly increases in the striatum of 6-OHDA rat (*n* = 4) and MPTP monkey (*n* = 3) models of PD. * denotes a significant difference (Student *t*-test ; *p* < 0.05).

Experimental parkinsonism and PD induced an increase in immunostaining intensity of GFAP-S-100β in both the striatum (Figure 1A) and GP (Figure 1B), whatever the considered species (mouse, rat, macaque and human) or modeling methodology across species. The main cellular processes are thicker and the number of primary processes leaving the soma is increased as well. Parkinsonism-associated astrocytes display a larger soma (up to 20% larger) in both the 6-OHDA rat (*p* < 0.001, Figure 2A) and the MPTP-treated macaque (*p* < 0.001, Figure 2A). Such hypertrophy of GFAP-containing cellular processes is characteristic of reactive astrocytes.

Longer processes once activated (Figure 2A) are however observed in primates rather than in the rat (Figure 2A), as reported as well in humans. When visualized by antibodies against intermediate filament protein GFAP, these thicker processes in macaque appear longer (Figure 2A) than in the rat (Figure 2A) because they can be followed over greater distances.

Two astroglial cell populations could be distinguished by their morphology: a first group featuring astrocytes with high staining intensity, a few large and long processes and a second group with low staining and thin processes (Figure 2A). Activated astrocytes in MPTP-treated macaque or in human PD show very long glial processes with some enlargements, more or less regularly arranged along the length (Figure 2A). In the MPTP-treated macaque, the longest processes vary in length from < 90 to > 300 μm with an average length of 190 mm ± 8 μm based on GFAP immunolabeling (*n* = 150 processes, measured from *n* = 3). Previous report in human cortex had noted such long processes associated with an increased complexity (branching) (Oberheim et al., 2009), an observation similar to ours in MPTP-treated macaque and in human PD that is not seen in the 6-OHDA-lesioned rat model.

### Increase in number of astrocytes in experimental parkinsonism

The above results show that the intensity of labeling increases in experimental parkinsonism and in human PD, likely reflecting the activation of astrocytes with larger somas and longer/thicker processes. We then counted the number of astrocytes in the experimental parkinsonism (reserpine-treated mouse, 6-OHDA-lesioned mouse and rat, MPTP-treated macaque) and in human PD both in the striatum and the GP (GPe in primates). Whatever the method for generating experimental parkinsonism, both acute DA depletion (reserpine) and chronic nigrostriatal degeneration (6-OHDA, MPTP) induce a significant increase in the number of striatal astrocytes (*p* < 0.05, Figure 2B). In human PD, there is only a trend for an increase in the number of astrocytes in the caudate nucleus (Figure 2B), a result likely to be the consequence of the tissue quality, the post-mortem time and more importantly the chronic exposure of patients to a number of drugs with unknown impact upon astrocytosis. Experimental data suggest that L-dopa, the reference treatment of PD likely used by patients, does not normalize the number of astrocytes (data not shown) but PD patients receive polytherapies for their parkinsonism and other age-related conditions. Pattern is however less obvious in the GP with a significant increase in the reserpine-treated mouse and the 6-OHDA-lesioned rat but not in the 6-OHDA-lesioned mouse, the MPTP-treated macaque or human PD. Of note however is the consistently higher number of astrocytes in the GP than in the striatum in animals and in humans (Figure 2B).

### Increase in astrocyte area in experimental parkinsonism

To determine whether hypertrophy of cellular processes affects the area of tissue reached by reactive astrocytes, we compared the area accessed by reactive astrocytes vs. nonreactive astrocytes (GFAP immunolabeling) in the striatum and the GP of 6-OHDA-lesioned rats and MPTP-treated macaques. The tri-dimensional reconstruction is illustrated for these two models as well as for human PD (Figure 2C left), unraveling that astrocytes occupy a larger striatal volume in both experimental parkinsonism and human PD. There is a significant increase in the astrocytic area in the striatum of 6-OHDA-lesioned rats and MPTP-treated macaques (*p* < 0.001, Figure 2C) and in the GP of the 6-OHDA-lesioned rats (*p* > 0.001, Figure 2C). Semi-quantitative measurement in human PD (limited by the human material quality and the unknown duration and dose exposure to DA replacement therapy in their lifetime) shows a 17% increase as well in astrocyte area. The very large increase in striatal volume occupied by astrocytes in parkinsonism is likely primarily due to the lengthening and the thickening of astrocyte processes since the enlargement of soma size remains modest (though significant, Figure 2A), enabling the astrocytes to modulate excitatory striatal transmission in parkinsonism. Such result contradicts previous observations collected in the denervated hippocampal region and the electrically-injured cerebral cortex (Wilhelmsson et al., 2006) suggesting that despite the increased thickness of astrocytic cellular processes in those two areas, astrocytes did not occupy a greater volume of tissue and did not alter the action radius of reactive astrocytes in the hippocampus. Morphological and functional responses of astrocytes likely depend on the type of insult and on the cerebral region as the astrocytes in DA-depleted striatum occupies a larger area.

### Connexin expression is modulated in experimental parkinsonism

**Figure 3:**
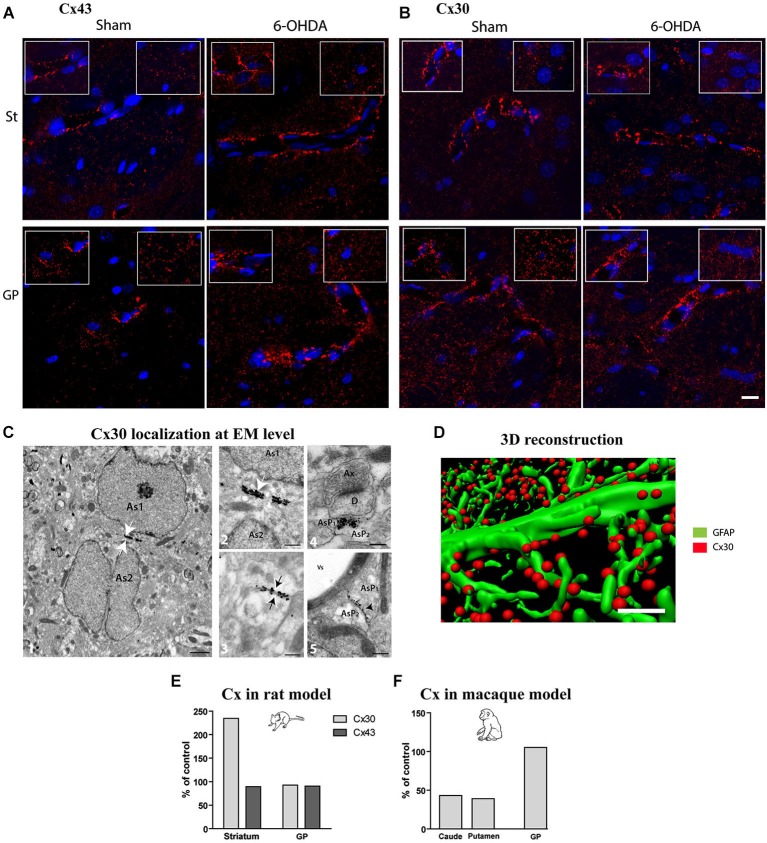
**Qualitative and quantitative assessment of connexin 30 and 43 expression in rat and monkey models of PD**. Connexin 43 **(A)** and 30 **(B)** distribution at cellular level in the striatum (top row) and GP (bottom row) of sham and 6-OHDA rat model of PD. Images correspond to the Z projection of six deconvolved 1 μm optical sections through section, red staining for Cx and blue for nucleus. Insets point out representative Cx immunolabeling around vessels (left) or in neuropil at distance from vessels (right) (scale bar = 10 μm). **(C)** 1-Representative electron micrographs showing Cx30 localization (scale bar = 1 μm); 2-symmetrically labeled gap junction between two astrocytic soma (white arrow; As = astrocyte) (scale bar = 0.5 μm) ; 3- symmetrically labeled gap junction between two astrocytic processes (black arrow; AsP = astrocytic process) (scale bar = 0.5 μm); 4-symmetrically labeled gap junction between two astrocytic processes close to asymmetrical synapse (scale bar = 0.5 μm); 5-asymmetrical labeled gap junction between two astrocytic processes (scale bar = 0.5 μm). **(D)** Illustration of quantification methodology of connexin based on double immunolabeling. GFAP immunostaining appears in green while Cx 30 immunostaining appears in red (scale bar = 2 μm). **(E)** Connexin Cx30 and Cx43 quantification in the 6-OHDA rat and **(F)** in MPTP monkey models of PD expressed in percentage of levels measured in control animals.

**Figure 4:**
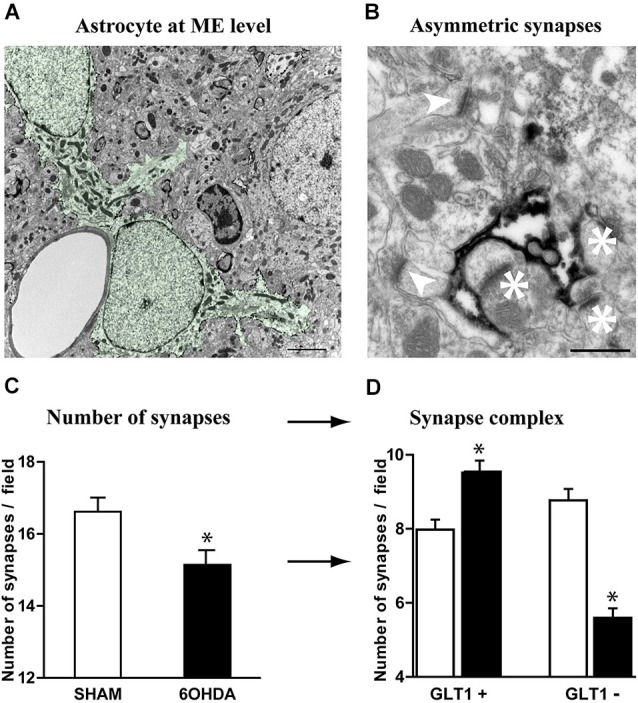
**Astrocytes further contacts asymmetric striatal synapses after dopamine depletion. (A)** Electron micrograph of GLT1 immunolabeled striatal astrocytes colored in green (pseudocolor image NHI Image J) (scale bar = 2 μm). **(B)** GLT1-positive astrocytic processes surrounding (asterisk) or not surrounding asymmetric striatal synapses (arrowhead) (scale bar = 0.5 μm). **(C)** A significant decrease in the number of asymmetric synapses is observed in 6-OHDA rat striatum compared with control (*n* = 4 animals per group; Student *t*-test; *p* < 0.05). **(D)** Such decrease however decomposes in a significant increase in GLT1+ asymmetric synapses and a dramatic decrease in the number of GLT1− asymmetric synapses in the 6-OHDA rat compared to controls (*n* = 4 animals per group; one-way ANOVA followed by Bonferroni; *p* < 0.05).

Considering that experimental parkinsonism induces a large increase in astrocytic volume and that astrocytes are organized into functional networks through interconnection via intercellular GJs composed of Cx (Giaume and Mccarthy, 1996; Nagy et al., 1999; Dermietzel et al., 2000), we sought to determine if expression pattern and density of Cx30 and Cx43 connexins were affected by DA depletion. Distribution of Cx43 (Figure 3A) and Cx30 (Figure 3B) is heterogeneous with higher expression of both Cx in the GP than in the striatum. Cx30 (Figure 3B) and Cx43 (Figure 3A) stainings in the striatal and GP neuropil of 6-OHDA-lesioned rats were differentially affected. Immunostaining greatly strengthened around vessels for both Cx43 and Cx30 (Figures 3A,B), suggesting an increased metabolic coupling. Electron microscopy observations confirmed the localization of Cx30 along the inner gap junctional membranes of astrocytic processes (Figure 3C). Cx30 expression is particularly abundant between neighboring astrocytes (Figure 3C-2), between astrocytes processes (Figure 3C-4-5), in astrocytic processes sheathing chemical synapses (Figure 3C-3) as well as on the astrocytic end feet surrounding blood vessels (Figure 3C-5).

Quantitative assessment of connexin expression was achieved using immunohistochemical labeling followed by 3-D reconstruction and automated quantification of the number of Cx positive puncta, a measurement performed strictly in neuropil area devoid of vessels (Figure 3D). The 6-OHDA-lesioned rat displayed an increased density of Cx30 in the striatum (Figure 3E) but not of Cx43 and none of the two Cx in the GP (Figure 3E). The MPTP-treated monkey however showed a decrease in Cx30 levels in the striatum but not in the GP (Figure 3F). Altogether, these data suggest that, while astrocytosis is massive in experimental parkinsonism, it is not associated with an increased coupling between astrocytes but might be associated to an increased metabolic coupling. However, the functional impact of alterations in connexin expression on metabolite trafficking through astrocytic networks has never been directly assessed in any neurodegenerative diseases.

### Increased astrocytic “control” of striatal asymmetric synapses in Parkinsonism

Tripartite synapses, i.e., the structure consisting of pre- and post-synaptic elements of the synapse and an associated astrocyte process (Perea et al., 2009; Parpura et al., 2012; Santello et al., 2012), are a marker of increased control of astrocytes upon synaptic transmission. The loss of nigrostriatal DA neurons results in medium spiny neuron spine retraction, and the loss of glutamatergic asymmetric synapses in rodent (Ingham et al., 1989; Day et al., 2006; Schuster et al., 2009; Zhang et al., 2013; Suárez et al., 2014) and primate (Scholz et al., 2008; Villalba and Smith, 2011) models of PD. Given the dramatic changes affecting astrocytic morphology in striatum of experimental parkinsonism and human PD, we investigated at electron microscopy level the evolution of the relationship between the astrocytic processes and the asymmetric synapses representing the corticostriatal and thalamostriatal glutamatergic input, using immunohistochemical methods (immunogold or peroxidase) with antibodies against the glutamate transporter GLT1 as astrocytic plasma membrane marker. GLT1 is predominantly expressed in astroglia, found both in astrocytic cell bodies and in the smallest ramifications of the astrocytes close to synapses and around blood vessels (Chaudhry et al., 1995; Lehre et al., 1995) and is heavily expressed in striatum (Lehre et al., 1995; Figure 4A). We considered as GLT1-positive asymmetric synapses only those that were GLT1 immunoperoxidase-labeled on the edge of axon-dendrite interfaces (Figure 4B). Quantitative analysis unraveled a significant reduction (9%, *p* < 0.05) in asymmetric synapses in 6-OHDA rats compared with controls (Figure 4C) in agreement with literature (Ingham et al., 1989; Day et al., 2006; Scholz et al., 2008; Schuster et al., 2009; Villalba and Smith, 2011; Zhang et al., 2013). Asymmetric synapses surrounded by GLT1-positive astrocytic processes were however increased in 6-OHDA rats (*p* < 0.05; Figure 4D) while those not surrounded by astrocytic processes dramatically decreased (*p* < 0.05; Figure 4D). These data suggest that, while the nigrostriatal lesion induces a loss in striatal asymmetric synapses, the astrocytic control over striatal glutamatergic transmission increases in parkinsonism.

## Discussion

The study of the PD-related modifications in astrocytic morphology unraveled that DA depletion or denervation is accompanied in the striatum by a dramatic increase in astrocyte number (+20%), in the astrocyte volume (+60%) and in the number of glutamatergic synapses contacted by GLT1+ astrocytic contacts (+15%). These unequivocal data support our hypothesis of a dramatic regulation of striatal astrocytosis in position for (dys) regulating excitatory striatal input, i.e., arising from cortex and thalamus.

The present work, undertaken in four animal models of PD as well as in human PD brains, highlights that in response to DA denervation or PD, astrocytes undergo characteristic phenotypic changes known as reactive astrogliosis, a non-specific but highly characteristic response that involves various morphological and molecular changes. Whatever the animal model of PD or human parkinsonism, we observe in both the striatum and GP, an enlargement of astrocyte cell body, a remodeling of processes and an increase in astrocyte number that are indications of reactive gliosis in agreement with previous studies (Muramatsu et al., 2003; Dervan et al., 2004; Gomide and Chadi, 2005; Même et al., 2006; Henning et al., 2008). The grade of astrogliosis appears however to be directly related to the method by which the dopaminergic lesion was induced. Reactive astrogliosis is actually not a simple all-or-nothing phenomenon but, rather, a finely gradated continuum of molecular, cellular and functional changes ranging from subtle alterations in gene expression to scar formation. Dysfunction of either astrocytes or the process of reactive astrogliosis is emerging as an important source of potential mechanisms that might contribute to, or play primary roles in, a host of CNS disorders via loss of normal or gain of abnormal astrocyte activities.

Our observations show in striatum and GP morphological differences between primate and rodent astrocytes with an increased complexity and size of primate protoplasmic astrocytes, an inter-order feature previously noted for cortical astrocytes (Oberheim et al., 2009).

Individual astrocytes play a major role in synaptic physiology by controling a large number of synapses, in part through gliotransmission, or glutamate and K+ uptake (Nedergaard and Verkhratsky, 2012). However, another key property of astrocytes is their extensive network communication via intercellular GJ channels, formed by Cx. These Cx are poorly selective channels allowing direct cytoplasmic exchange of a variety of small molecules like ions, second messengers, neurotransmitters or energy metabolites (Giaume and Mccarthy, 1996; Dermietzel et al., 2000; Giaume and Theis, 2010). The two main Cx expressed in astrocytes are connexin 43, present from embryonic stages to adulthood, and connexin 30, expressed after postnatal day 10 (Nagy et al., 1999). GJ channels mediate the formation of large cellular ensembles reaching millimeter size in different brain regions, encompassing hundred to thousands of astrocytes (Ball et al., 2007; Giaume and Theis, 2010). Nevertheless, these connections can be selective and preferential since adjacent astrocytes are not always functionally connected (Houades et al., 2006) which may result from heterogeneous expression of Cx in astrocytes, or to short-term regulation of astroglial GJ permeability. Classically, a reactive gliosis is associated with modifications in the expression and function of these astrocytic Cx (Giaume et al., 2010, 2013). Our study shows a differential modulation in Cx expression in the rat and monkey model of PD preventing any final conclusion to be drawn about quantitative changes within a given structure. Interestingly however, both species showed a higher expression of both Cx in the GP than in the striatum (Nagy et al., 1999), a feature that was maintained in the parkinsonian condition. The consolidation of astroglial networks in the GP may favor specific neuroglial interactions. Indeed, this might contribute to local and precise processing of neuronal signals, favoring circuit independence by restricting information transfer to distal neurons, belonging to the GP. This region-dependent organization of astroglial networks may also directly influence how distant neurons with high-energy needs, within a specific network, are supplied with metabolites taken up by perivascular astrocytes.

We found an increased expression of both Cx30 and Cx43 in perivascular astrocytic endfeet and delineation of blood vessel walls in rat and monkey models of PD. The functional connectivity of perivascular astrocytic networks has been hypothesized to contribute to neurometabolic coupling since astroglial Cx mediate an activity-dependent glucose distribution through intercellular astroglial networks (Rouach et al., 2008). Astrocytes actually define the local availability of energy substrates by regulating blood flow. Subsequently, in order to efficiently reach distal neurons, these substrates can be taken up, and distributed through networks of astrocytes connected by GJ, a process modulated by neuronal activity.

We highlighted that Cx show differential changes in the two animal models of PD, thus further studies are needed to sort out this question. While the reactive astrogliosis occurs following acute (reserpine) or chronic (6-OHDA, MPTP, human PD) DA depletion, Cx expression regulation, and hence, physiological function, might well takes time to establish and the regulatory mechanisms may be different, thereby explaining the differences between the 6-OHDA rat (3 weeks of depletion) vs. the MPTP monkey (few months of depletion).

A striking observation of the study is that asymmetric excitatory cortico- or thalamo-striatal synapses decrease in 6-OHDA rats compared with controls (Figure 4C), a result in agreement with literature in the 6-OHDA rat (Ingham et al., 1989; Day et al., 2006; Scholz et al., 2008; Schuster et al., 2009; Donnelly et al., 2013; Suárez et al., 2014) and in the MPTP-treated macaque monkey (Villalba and Smith, 2011). Further investigations of vGLUT1 and vGLUT2-labeling of asymmetric synapses would help defining if the cortico-striatal or thalamo-striatal pathways, respectively (Raju et al., 2008), are affected comparably or differentially by an increased apposition of GLT1+ astrocyte feets. In parkinsonism, we have clearly identified changes in astrocytic morphology which are accompanied by processes reorganization at the level of asymmetric synapses.

A major role of astrocytes is the uptake of neurotransmitters allowed by the presence of astrocytic transporters which remove most of the glutamate that spills over the synaptic cleft (Kullmann and Asztely, 1998; Danbolt, 2001; Goubard et al., 2011). In rodents, GLT1, highly expressed in astrocytes, is responsible for the largest proportion of total glutamate uptake in the forebrain (Rothstein et al., 1996; Tanaka et al., 1997). Astrocytes express also, at a lower level, glutamate-aspartate transporter (GLAST). The astrocyte to neuron lactate shuttle (ANLS) hypothesis states that enhanced neuronal activity at glutamatergic synapses stimulates the uptake of glutamate, which is co-transported with Na+ in astrocytes via GLT1 or GLAST. Restoration of the Na+ gradient by the Na/K ATPase pump consumes ATP, whose levels are recovered by an enhanced uptake and glycolysis of glucose in astrocytes. Glycolysis in astrocytes leads to rapid production of lactate, which is then transported to neurons, thereby fueling their energy needs (Pellerin and Magistretti, 1994). A very tight cooperation between astrocytes and neurons for glucose oxidation in conditions of enhanced neuronal activity is now well documented, allowing to a enhanced neuronal activity to be translated into an increased uptake of glucose in the active regions.

We highlighted that, while the nigrostriatal lesion induces a loss in striatal asymmetric synapses, the astrocytic control over striatal glutamatergic transmission increases in PD. Such a phenomenon can affect the extracellular space and likely regulates metabolic exchanges at the tripartite synapse, placing such synaptic organization in key position for playing a role in expression of cortico-striatal transmission-related symptoms (Goubard et al., 2011). In a seminal study, Goubard et al. showed that glutamate and GABA uptake by astrocytes plays a key role in the corticostriatal information processing, thereby heavily participating in the filtering of corticostriatal information (Goubard et al., 2011). One can thus hypothesize that such filtering is impaired in PD-like DA-depleted situations and that astrocytosis plays a yet unacknowledged role in PD pathophysiology.

Our results unravel a dramatic regulation of striatal astrocytosis supporting the hypothesis of a key role in (dys) regulating excitatory cortico-/thalamo-striatal transmission. Such fine-tuning of excitatory transmission must now be investigated electrophysiologically. Astrocytes and their various properties could thus represent an interesting therapeutic target for symptomatic management in PD.

## Author contribution statement

Erwan Bezard designed and organized the experiments; Giselle Charron, Evelyne Doudnikoff, Marie-Helene Canron, Qin Li, Sebastien Marais, Jérôme Baufreton, Anne Vital and Stéphane H. R. Oliet performed experiments; Giselle Charron, Evelyne Doudnikoff and Erwan Bezard analyzed the data; Giselle Charron, Evelyne Doudnikoff, Céline Véga, Stéphane H. R. Oliet and Erwan Bezard wrote the paper.

## Disclosure statement

The Université de Bordeaux and the Center National de la Recherche Scientifique provided the infrastructural support. The funders had no role in study design, data collection and analysis, decision to publish, or preparation of the manuscript. Appropriate approval and procedures were used concerning human subjects and animals.

## Conflict of interest statement

The authors declare that the research was conducted in the absence of any commercial or financial relationships that could be construed as a potential conflict of interest.
